# Manifestations of Anti-Black Racism and Worry About Pregnancy and Birthing While Black: A Cross-sectional Secondary Analysis of Giving Voice to Mothers

**DOI:** 10.1007/s40615-025-02461-2

**Published:** 2025-05-06

**Authors:** Dorian S. Odems, Erica Czaja, Saraswathi Vedam, Na’Tasha Evans, Barbara Saltzman, Karen A. Scott

**Affiliations:** 1 Department of Human Ecology, University of California, Davis, One Shields Ave, Davis, CA 95616 USA; 2 Department of Political Science, College of the Holy Cross, 1 College St, Worcester, MA 01610 USA; 3 Birth Place Lab, Division of Midwifery, University of British Columbia, E416 Shaughnessy (Mailbox 80), 4500 Oak Street, Vancouver, BC V6H 3 N1 Canada; 4 College of Medicine, University of Kentucky, Lexington, KY USA; 5 College of Health and Human Services, University of Toledo, 3000 Arlington Ave, MS 1027, , Toledo, USA; 6 Birthing Cultural Rigor, LLC, 3820 Charlotte Ave, Ste 146-23, Nashville, TN 37209 USA

**Keywords:** Black women, Worry, Pregnancy, Birth, Obstetric racism, Structural racism

## Abstract

**Introduction:**

Pregnancy and childbirth traditionally bring worry or a sense of anxiety and distress, particularly among Black women that face historical and contemporary anti-Black racism. We employed two frameworks to assess manifestations of anti-Black racism, *structural racism* and *obstetric racism*, as predictors of worry about pregnancy and birth within the Black reproducing community.

**Methods:**

In a secondary cross-sectional analysis, we analyzed data from Black women in the Giving Voice to Mothers study who completed all relevant items (*n* = 260). We conducted descriptive analyses and logistic regression models to explore how worry about pregnancy and birth for the Black reproducing community varies with experiences of obstetric racism and different manifestations of structural racism.

**Results:**

Approximately 71% of the sample worried about pregnancy and birth for themselves and their community. Black women who experienced obstetric racism were statistically significantly more likely to be worried about pregnancy and birth experiences compared to Black women who did not. Furthermore, when structural racism was manifested and measured as hidden resources, among Black women reporting fewer pregnancy and birthing care options for women of color, those who experienced obstetric racism during care were 15.6 times more likely to worry about pregnancy and birthing experiences than those who did not (OR 15.667; 95% CI 1.348–182.058).

**Conclusion:**

The findings demonstrate the complexity of racialized harm enacted against Black women during the perinatal period and underscore the ways in which obstetric racism and contexts of structural racism powerfully shape the meaning and subsequent emotional impact of pregnancy and birthing while Black.

**Supplementary Information:**

The online version contains supplementary material available at 10.1007/s40615-025-02461-2.

## Introduction

Despite advancements in reproductive technology and obstetric patient safety bundles, health systems and hospitals in the United States (U.S.) permit the worsening of perinatal health outcomes among Black mothers and their infants in comparison to non-Black mothers and their infants [1–5]. Public health and clinical approaches to address adverse perinatal outcomes, particularly among Black women, uphold mother-blame narratives, defined as holding women exclusively responsible for adverse perinatal outcomes impacting them and their children [6–8]. For example, The Joint Commission’s Speak Up Campaign acknowledges that patient safety is threatened by racism and provides information for patients to “speak up” and report discriminatory care practices [9]. Therefore, the onus to mitigate mistreatment during care is on the pregnant or birthing patient instead of the hospital system, which creates an unsafe environment for birth, reinforcing mother-blame narratives. Scholars from public health, social sciences, and humanities challenge maternal child health researchers, advocates, clinicians, and practitioners to stop attributing inequities in perinatal health outcomes on race, personal characteristics, or “cultural” behaviors, and instead focus on research and mitigation efforts to counteract the effects of exposure to racism [6, 10–14]. Clinicians, perinatal quality improvement initiatives, and public health practitioners in the U.S. have traditionally focused on outcome measures, such as preterm birth, infant or maternal mortality, low birth weight, and maternal morbidities, as indicators of health and quality of care without accounting for concomitant patient experience outcomes. Notably, transdisciplinary scholars now emphasize that perinatal outcomes are inadequate indicators of quality, patient safety, and hospital performance if they do not include metrics on the care experience. In 2016, the World Health Organization published its *Standards for Improving Quality of Maternal and Newborn Care in Health Facilities*—a landmark publication that brought global attention to quality standards and the measurement of adverse care experiences [15]. However, in the U.S., the focus remains on survival through quality improvement bundles and evaluating traditional perinatal outcomes such as maternal morbidity. Standard perinatal outcomes often reflect Black women's responses to care shaped by generations of reproductive control, dominance, and injustice [16, 17]. Likewise, transdisciplinary scholars call for a new patient safety paradigm that extends beyond “being safe” as defined by quality and safety professionals toward emotional safety and “feeling safe” [16]. Currently, two patient-driven obstetric quality measures exist that prioritize emotional safety as defined through patient experiences and community wisdom, using obstetric racism as an exemplar: the SACKRED Birth framework and the Patient-Reported Experience Measure of Obstetric Racism©, (PREM-OB Scale®) [18, 19]. Public health, quality improvement professionals, and patient safety experts in the U.S. have yet to adequately prioritize emotional safety as a measure of patient safety and patient experience as an indicator of quality despite evidence that patient experience is positively associated with patient safety and clinical effectiveness and that women identify feelings of safety as essential to the perinatal experience [20–22]. In this paper, we respond to recent calls for scientific and cultural advancements in the ethical, epistemological, theoretical, and methodological approaches to naming and measuring quality and safety by applying Black women–defined theories that include the historical contextualization and contemporary implications of U.S. chattel slavery, colonization, misogynoir, gender oppression, and anti-Black racism, to Black women’s experiences of care [4, 6, 16, 17, 23–28].

Obstetric violence is a form of gender-based violence enacted against women and reproducing capable people during or related to pregnancy, childbirth, and postpartum by any personnel within medical institutions, resulting in reproductive control and dominance during care provision, treatment, diagnosis, and decision-making, and concomitant loss of patient autonomy, safety, and dignity while seeking perinatal care [3, 29]. There is varying language across countries and in the literature for defining, categorizing, and measuring obstetric violence [30–32]. Researchers in North America have adopted the term “mistreatment” as a more inclusive term to describe health system conditions and individual behaviors (i.e., obstetric violence), whether intentional or unintentional, and created a typology of mistreatment for measurement and policy development [30]. One in six women in the Giving Voice to Mothers study (GVtM) reported experiencing mistreatment during pregnancy and birthing care, with Black women and non-Black women of color reporting higher rates of mistreatment (23.8%) than white women (14.1%) [33]. Other studies have also reported on the healthcare experiences of women who identify as Black and non-Black people of color and have documented how racially marginalized groups are mistreated and given inadequate care [34, 35]. In a cross-sectional study utilizing GVtM data, researchers assessed experiences of non-consented perinatal procedures among Black and non-Black people of color. Study findings revealed that Black women were more likely to report procedures being performed on them without their consent than those racialized as white [36]. These studies suggest that health systems enact distinct acts of obstetric violence and mistreatment against Black women and birthing people during perinatal care that demand further investigation if they are to be properly understood and prevented.

Obstetric violence and mistreatment threaten the health of women, pregnant, and birthing people worldwide; however, these frameworks lack an intersectional lens that includes an interrogation of both race and gender as modes of knowledge production in understanding, examining, and explaining Black women and people’s expectations and experiences [37, 38]. In the U.S. context, examining and explaining perinatal care outcomes and experiences among Black women and reproducing capable people through obstetric violence or mistreatment offer a gendered analysis without the historical contextualization and contemporary implications of U.S. chattel slavery, plantation colonization, anti-Black racism, misogynoir, and anti-Black gender oppression. Consequently, the terms “obstetric violence” or “mistreatment” obfuscate the distinct medical experiences of Black pregnant and birthing people [37]. Dr. Dána-Ain Davis defines these experiences within the phenomenon and analytical framework of *obstetric racism*, positioning obstetric racism as the intersection of medical racism and obstetric violence [3]. Davis developed six domains of obstetric racism that can be identified in the medical encounters of Black women that track along histories of anti-Black racism and eugenics: diagnostic lapses; neglect, dismissiveness, or disrespect; intentionally causing pain; coercion; ceremonies of degradation; and medical abuse [3, 39–41]. Enacted throughout the social and clinical encounters for any pregnancy-related condition with healthcare personnel regardless of race, gender, or profession, obstetric racism serves as a threat to positive birth outcomes, reproductive justice, quality, and patient safety among Black women and their given and chosen kin [2, 4, 17, 37, 42].

Obstetric racism refers to the beliefs and practices within health systems that impede on the autonomy, human rights, and liberation of Black women during care, while structural racism encompasses a set of laws, policies, and practices—both within and beyond health systems—that unfairly disadvantage Black women and other racially marginalized groups while unfairly advantaging others. [13, 14, 43]. Maternal and child health scholars have explored the relationships between measures of structural racism and perinatal health outcomes, including the utilization of geographical, judicial, and neighborhood-level indicators of structural racism to assess these relationships [44–49]. However, these definitions and measures of structural racism are not developed through the lived experience of Black women and limit our understanding of the harms experienced by the Black reproducing community. Notably, Chambers et al. developed a novel conceptual framework of *Structural racism from the perspectives of Black women across the reproductive lifespan* to name and define the impact of structural racism on Black women’s inequitable perinatal outcomes. Black women who were preconception, pregnant, or postpartum identified nine domains of structural racism using a grounded theory approach: (1) *negative societal views*, (2) *housing*, (3) *medical care*, (4) *law enforcement*, (5) *hidden resources*, (6) *employment*, (7) *education*, (8) *community infrastructure*, and (9) *policing black families* [50]. Interestingly, policing of Black families and hidden resources were themes that emerged only from the input of pregnant and postpartum women [50]. Child protective services, referred to as the family policing system, disproportionately surveil Black women and families during and after pregnancy; a punitive system rooted in anti-Black racism and traced back to enslavement, intentionally separating Black children from their mothers and placing them in the care of former enslavers or “better-suited” White families [51]. In a study of Black pregnant and postpartum women who were incarcerated during pregnancy, used illegal substances, and had a history of family policing, many described acts of obstetric racism, inhumane treatment while incarcerated, internalized mother-blame narratives, and even the inability for some Black women to identify how structural racism impacts their lives while simultaneously describing its effects [52]. As a collective, the nine domains of structural racism across the reproductive lifespan illuminate how Black women encounter harmful systems and structures, both within and outside of healthcare settings, that persistently subjugate them and threaten the health of Black women and their families. Combining *obstetric racism* [3, 4, 39–41] and *structural racism across the reproductive lifespan* [50] provides a nuanced and novel understanding of beliefs, practices, policies, processes, programs, and procedures across various systems and structures exclusive to the field of obstetrics and more broadly within and beyond health systems that include family policing systems, law enforcement, and other social and structural drivers (e.g., housing, community infrastructure, education).

The reduction in structural and systemic barriers, burdens, and risks to seeking perinatal care, such as obstetric racism and structural racism, requires a blueprint. In 1995, Women of African Descent for Reproductive Justice defined reproductive justice as (1) the human right to maintain personal autonomy, (2) the right not to have a child, (3) the right to have a child, and (4) the right to parent children in safe and healthy environments [53]. As noted in the GVtM study descriptive report, Black women were disproportionately worried about their experiences of pregnancy or giving birth [54]. In this paper, we define worry as a feeling of anxiety, mental agitation, or distress as a result of concern about anticipating or experiencing a difficult event or interaction related to pregnancy and childbirth [55]. Having deep worry about pregnancy and birthing while Black threatens reproductive justice. Black women’s worries about pregnancy and birth may deter their desire to have a child or undermine their autonomy, agency, and self-efficacy in determining their pregnancy-related journey. Ross and Solinger [56] state, “This requires a safe and dignified context for these most fundamental experiences.” Acts of obstetric racism as defined by Davis [3, 4, 39–41] and structural racism across the reproductive lifespan as defined by Chambers et al. [50] not only strip Black women of their human right to personal autonomy but threaten one of the first opportunities to parent in a safe and healthy environment (i.e., childbirth). For example, reminiscent of the afterlife of slavery, clinicians may retaliate against Black women for “non-compliance” through forced parent–child separation after birth, denying opportunities for bonding and human milk feeding as a violation of birthing rights and patient safety [3, 4, 37]. Forced or coerced parent–child separation after birth usually occurs through physician, nurse, or social worker (system) initiated involvement of family policing systems, psychiatry, hospital security, or local law enforcement. These unjust and punitive acts by hospital personnel are defined as ceremonies of degradation within the obstetric racism framework [3, 39, 41] and policing of Black families within structural racism across the reproductive lifespan [50]. Both frameworks offer clear domain names, meanings, and manifestations that violate one or more of the three tenets of reproductive justice, specifically impacting Black women and people during care provision and, therefore, embody acts of reproductive injustice within and outside of the health system during pregnancy and postpartum. For clarity, Table 1 lists both frameworks and their respective domains. There is no inherent meaning or significance in the order of domains or in the connections between each domain within their respective frameworks. Definitions for each domain of both frameworks are provided in Supplementary Material 1.

**Table 1:** Theoretical Frameworks and Respective Domains

	Dimensions of obstetric racism (domains and responses)	Domains of structural racism across the Black reproductive lifespan
1	Neglect, dismissiveness, or disrespect	Negative societal views
2	Medical abuse	Housing
3	Coercion	Medical care
4	Intentionally causing pain	Law enforcement
5	Ceremonies of degradation	Hidden resources
6	Diagnostic lapse	Employment
7	Racial reconnaissance	Education
8	Resistance	Community infrastructure
9	Refusal	Policing Black families

Definitions for each domain can be found in Supplementary Material 1

Heightened worry for pregnancy and birthing experiences while Black in the U.S. is justified considering the stark racial inequities in perinatal outcomes and extensive exposure to various types and levels of anti-Black racism inside and outside of perinatal care [2–4, 36, 47, 57–63]. Taken together, the higher reports of mistreatment, reduced autonomy, non-consented care, and worry among Black women in GVtM specifically signaled the need for a more complex intersectional analysis of the data to examine their experiences and potential intersectional solutions to prevent and mitigate violations of quality and patient safety [33, 36, 54].

Using Davis’ obstetric racism [3, 4, 39–41] and Chamber et al.’s structural racism across the reproductive lifespan [50] to understand, analyze, and interpret acts of reproductive injustices among Black women during pregnancy and postpartum, this study aims to answer three research questions: (1) What is the prevalence of worry about pregnancy and giving birth for themselves and their community among Black women who gave birth or were pregnant between 2010 and 2016 in the U.S.?; (2) How do Black women’s experiences of obstetric racism influence their worries about pregnancy and birth for themselves and their community?; and (3) How do contexts of structural racism across the Black reproductive lifespan moderate the relationship between experiences of obstetric racism and worry about pregnancy and birth for themselves and their community?

## Methods

### Data Source

This secondary data analysis utilized the Giving Voice to Mothers (GVtM) survey dataset. GVtM is a national cross-sectional survey that aimed to capture care experiences during and after pregnancy and childbirth in the U.S, from 2010 to 2016 (*n* = 2700). This survey was developed between 2014 and 2016 with extensive engagement and contributions by service users (mothers), clinicians, community health service leaders, providers, and researchers [54]. GVtM is the first U.S. study to use quantitative indicators created by service users to describe pregnancy and birthing care among those who had a community or hospital birth [54]. The GVtM study was administered electronically, and informed consent was obtained from all respondents. GVtM respondents were recruited by community partners who provided clinical or community health services via snowball sampling, social networking, listservs, referrals from parents and consumer advocacy groups in various states, and in-person venues where parents convene [36, 54]. Though the survey was not drawn from a nationally representative sample, the convenience sample included respondents from every state, Washington D.C., and Puerto Rico. In addition, respondents identifying racially as Black comprised 14% of the total sample (*N* = 380). The research reported herein utilized data from this subsample of Black women exclusively by removing missing data for all variables of interest, resulting in a sample size of *n* = 260. The first author’s university institutional review board reviewed the study methods and granted an exemption for human subjects review.

The GVtM dataset was selected for a secondary analysis because this survey was developed to capture the experiences of pregnancy and birth in the U.S. and included numerous validated measures of discrimination and racism against women of color (e.g., adapted Perceptions of Racism Scale [64]) and mistreatment during care. Although it was not designed to exclusively understand Black women’s pregnancy and birthing experiences or designed to measure obstetric racism specifically, the data provide a valuable opportunity to address the overlapping and interlocking impact of structural racism and mistreatment, as a proxy of obstetric racism. In this paper, survey measures include the Mistreatment Index (MIST), aligned with the seven domains of Bohren’s typology, used to operationalize obstetric violence [30, 33]. For the purposes of the secondary data analysis, mistreatment serves as a proxy measure of obstetric racism. Additionally, survey items that align with Chambers et al.’s conceptual framework of structural racism are used to operationalize contextual exposure to other forms of anti-Black racism across the reproductive lifespan [50].

### Measures

#### Dependent Variable

##### Proxy Item to Measure Emotional Safety or “Feeling Safe”

Worry About Pregnancy and Birth. Respondents completed the following focal item, “Based on what I or members of my community have experienced, I am worried about our experiences of being pregnant, giving birth.” The responses were recorded as “not concerned” (coded 0) and “somewhat concerned” and “very concerned” (coded 1). Those who responded “don’t know” were excluded from the analyses.

### Independent Variables

#### Proxy Items to Measure Experiences of Obstetric Racism

Mistreatment. Respondents selected any issues or behaviors that applied to their pregnancy and birthing experience using the Mistreatment Index (MIST) [33]: (1) your private or personal information was shared without consent; (2) your physical privacy was violated; (3) health care provider(s) shouted at or scolded you; (4) health care provider(s) threatened to withhold treatment or to force you to accept treatment you did not want; (5) health care provider(s) threatened you in any other way; (6) health care provider(s) ignored you, refused to respond to your request for help, or failed to respond in a reasonable amount of time; (7) you experienced physical abuse; (8) or none of the above. In the analyses, mistreatment experiences were coded as a dichotomous indicator variable (1 if any mistreatment items were checked, 0 if not).

Pressure to Have Any Procedure During Labor or Delivery. Respondents reported if they felt pressure from a doctor or midwife to have any of the following interventions: medication to start labor, an epidural, continuous fetal monitoring, episiotomy, medicine for pain relief, a cesarean, and medication to speed up labor. The responses were recorded as “yes” or “no” for each intervention. In the analyses, pressure experiences were coded as a dichotomous indicator variable (1 if there was a “yes” response to any of the pressure items, 0 if not).

### Contextual (Moderator) Variables

#### Proxy Items to Measure Domains of Structural Racism Across the Black Reproductive Lifespan

Negative Societal Views Domain

“Welfare Queen.” Respondents selected the option that best describes how they feel about the following statement, “If a pregnant woman who is Black comes to a White doctor or midwife’s office, the office staff assumes that she is on welfare.” Responses were recorded as “Strongly Agree,” “Agree,” “Somewhat Agree,” “Somewhat Disagree,” “Disagree,” and “Strongly Disagree.” Respondents who indicated any level of agreement were coded as 1 (indicating a context of structural racism in this domain), and those who responded with any level of disagreement were coded as 0 (no context of structural racism in this domain).

#### Hidden Resources Domain

Women of Color Have Fewer Options for Care. Respondents selected the option that best describes how they feel about the following statement, “Pregnant women of color have fewer options for pregnancy and birth care.” Responses were recorded as “Strongly Agree,” “Agree,” “Somewhat Agree,” “Somewhat Disagree,” “Disagree,” and “Strongly Disagree.” Respondents who indicated any level of agreement were coded as 1 (indicating a context of structural racism in this domain), and those who responded with any level of disagreement were coded as 0 (no context of structural racism in this domain).

#### Education Domain

Educational Opportunity. Respondents selected the option that best describes how they feel about the following statement, “A white woman has more educational opportunities than a woman of color.” Responses were recorded as “Strongly Agree,” “Agree,” “Somewhat Agree,” “Somewhat Disagree,” “Disagree,” and “Strongly Disagree.” Respondents who indicated any level of agreement were coded as 1 (indicating a context of structural racism in this domain), and those who responded with any level of disagreement were coded as 0 (no context of structural racism in this domain).

We hypothesize a significant positive relationship between Black women’s experiences of mistreatment and pressure (i.e., proxy measures for obstetric racism) during perinatal care and subsequent worry about pregnancy and childbirth for themselves and their community (i.e., a proxy measure of emotional safety). Importantly, we consider the context of Black women’s perinatal experiences and further hypothesize that the relationship between experiences of obstetric racism and worry about pregnancy and birth will be stronger in contexts of structural racism across the Black reproductive lifespan, hereinafter referred to as [structural racism]. In other words, we expect exposure to contexts of structural racism to strengthen (and thus statistically increase the size and strength of) the relationship between experiences of obstetric racism and worry related to pregnancy and birthing while Black.

### Statistical Analyses

A descriptive crosstabulation table and chi-square tests were performed to describe sample characteristics and assess bivariate relationships between the dependent variable, independent variables, contextual moderator variables, and covariates (Supplementary Material 2). Then, logistic regression analyses were used to evaluate (1) the influence of mistreatment and pressure, as proxies of obstetric racism, on Black women’s worry about pregnancy and childbirth and (2) how this relationship may be moderated by contexts of structural racism in the domains of negative societal views, hidden resources, and education. Covariates and interaction terms for each proxy measure of structural racism were included in the final moderator analysis. Odds ratios (OR) and 95% confidence intervals (CI) were computed for each logistic regression model.

## Results

In Table 2, 71.5% of this sample reported some level of worry about “our experiences of being pregnant, giving birth,” based on what they or members of their community had experienced. Approximately 71% of the sample gave birth in a hospital setting and 29% in a community setting (i.e., home birth or a free-standing birth center). Additionally, 25% of the respondents reported racial concordance with their providers, and approximately 37% had doula support. Most of the sample (70.4%) had insurance other than Medicaid during their perinatal care, and 93.1% were born in the U.S. Within the sample, 24.2% reported some form of mistreatment during perinatal care, and 45.0% reported experiencing pressure to have one or more procedures during labor and delivery. Among those who expressed worry, approximately 31% reported mistreatment and about 48% reported pressure. However, among those who expressed no worry, only 6.8% reported mistreatment, and about 38% reported pressure. Chi-square tests revealed there was a significant association between reports of mistreatment and worry (*p* < 0.001) but no statistically significant association between reports of pressure and worry (*p* = 0.143). Additionally, among all covariates, no significant associations were observed with worry about pregnancy and birthing experiences.

**Table 2:** Worry about pregnancy and birthing experiences among Black women in GVtM by variables of interest and key covariates

Worry about pregnancy and birthing experiences				
	Overall	Not concerned *n* = 74 (28.5%)	Concerned *N* = 186 (71.5%)	
	*N* = 260	*n* (%)	*n* (%)	*p* value
Birth characteristics/covariates				
Birth Setting				0.268
Community birth	75 (28.8%)	25 (33.8)	50 (26.9)	
Hospital Birth	185 (71.2%)	49 (66.2)	136 (73.1)	
Income				0.457
< $30,000	53 (20.4%)	13 (17.6)	40 (21.5)	
$30,000–$49,999 $50,000–$69,999 $70,000–$99,999 $100,000 or more	56 (21.5%) 40 (15.4%) 35 (13.5%) 76 (29.2%)	12 (16.2) 15 (20.3) 11 (14.9) 23 (31.1)	44 (23.7) 25 (13.4) 24 (12.9) 53 (28.5)	
Insurance				0.380
Medicaid	77 (29.6%)	19 (25.7)	58 (31.2)	
Private or other	183 (70.4%)	55 (74.3)	128 (68.8)	
Born in the U.S				0.091
Yes	242 (93.1%)	72 (97.3)	170 (91.4)	
No	18 (6.9%)	2 (2.7)	16 (8.6)	
Patient–provider race				0.634
Racial concordance	65 (25.0%)	17 (23.0)	48 (25.8)	
Racial discordance	195 (75.0%)	57 (77.0)	138 (74.2)	
Doula support				0.459
Yes	97 (37.3%)	25 (33.8)	72 (38.7)	
No	163 (62.7%)	49 (66.2)	114 (61.3)	
Midwife provider				0.453
Yes	138 (53.1%)	42 (56.8)	96 (51.6)	
No	122 (46.9%)	32 (43.2)	90 (48.4)	
Independent variables				
Mistreatment during care				< 0.001
Experienced any mistreatment	63 (24.2%)	5 (6.8)	58 (31.2)	
Did not experience any mistreatment	197 (75.8%)	69 (93.2)	128 (68.8)	
Pressure during care				0.143
Experienced any pressure during care	117 (45.0%)	28 (37.8)	89 (47.8)	
Did not experience any pressure	143 (55.0%)	46 (62.2)	97 (52.2)	
Proxy measures of structural racism				
Negative societal views				0.001
Any level of agreement	133 (51.2%)	26 (35.1)	107 (57.5)	
Any level of disagreement	127 (48.8%)	48 (64.9)	79 (42.5)	
Hidden resources				< 0.001
Any level of agreement	181 (69.6%)	39 (52.7)	142 (76.3)	
Any level of disagreement	79 (30.4%)	35 (47.3)	44 (23.7)	
Education				0.110
Any level of agreement	187 (71.9%)	48 (64.9)	139 (74.7)	
Any level of disagreement	73 (28.1%)	26 (35.1)	47 (25.3)	

Broadly, most of the sample agreed with statements aligned with three domains of structural racism [50]: negative societal views (51.2%), hidden resources (69.6%), and educational opportunity (71.9%). When assessing bivariate relationships between each of these domains and worry, there was a statistically significant association between worry and negative societal views (*p* = 0.001) and between worry and hidden resources (*p* < 0.001), but there was no statistically significant association between worry and educational opportunity (*p* = 0.110).

Table 3 displays the results of a multivariable logistic regression model examining the statistical significance of the associations between the independent variables of interest (i.e., any mistreatment and pressure as proxy measures for experiences of obstetric racism) and the dependent variable, worry about pregnancy and birth. As hypothesized, Black women who experienced obstetric racism, as defined in this analysis as any mistreatment, were nearly 6.6 times as likely to be worried about pregnancy and birthing experiences compared to those did not experience obstetric racism or mistreatment (OR: 6.587; 95% CI 2.410–18.004). After adjusting for covariates, the relationship between obstetric racism (or any mistreatment) and worry about pregnancy and birthing experiences remained statistically significant (OR: 6.234; 95% CI 2.302–16.879). Contrary to our hypothesis, there was no statistically significant relationship between experiencing obstetric racism as defined by pressure during labor and delivery care and worry about pregnancy and birth.

**Table 3:** Results of multivariable logistic regression model of worry among Black women in GVtM

	Worry about pregnancy and birthing experiences	
Mistreatment during care	Crude odds ratio [95% confidence interval]	Adjusted odds ratio [95% confidence interval]
Experienced any mistreatment	6.587*** [2.410–18.004]	6.234*** [2.302–16.879]
Did not experience any mistreatment	Reference	Reference
Pressure during care		
Experienced any pressure during care	1.324 [0.674–2.602]	0.901 [0.494–1.643]
Did not experience any pressure	Reference	Reference

Included in the adj. model: community birth setting, income < $30,000, income = $30,000–$49,999, income = $50,000 = $69,999, income = $70,000–$99,999, insurance status, US born, racial concordance, doula support, and midwife provider

^*^p <.05. **p <.01. ***p <.001

Table 4 presents how the relationship between our proxy measures of obstetric racism (i.e., mistreatment and pressure) and worry is moderated by the context of structural racism. In the current secondary cross-sectional analysis, the contextual moderator variables are proxy measures for structural racism, measured as agreement or disagreement with statements that align with three of the nine domains of structural racism [50]. As hypothesized, we find an even stronger, statistically significant relationship between experiences of obstetric racism and worry about pregnancy and birthing within a context of “hidden resources” (structural racism domain characterized by women of color having fewer options for perinatal care) (OR 15.667; 95% CI 1.348–182.058). Notably, in this model, the coefficient for the direct effect of obstetric racism (as mistreatment) on worry is not statistically significant, meaning that in contexts without “hidden resources,” experiences of obstetric racism do not have a statistically significant influence on Black women’s worry about pregnancy and birthing while Black. No other statistically significant interactions were found with the other proxy measures of structural racism used in the analyses (i.e., negative societal views and education).

**Table 4:** Results of adjusted multivariable logistic regression model of worry about pregnancy and birth including interaction terms for proxy structural racism domains (hidden resources, negative societal views, and education) as potential moderators

Worry about pregnancy and birthing experiences						
	Hidden resources		Negative societal views		Education	
	Adj. odds ratio [95% CI]	*p*-value	Adj. odds ratio [95% CI]	*p*-value	Adj. odds ratio [95% CI]	*p*-value
Interaction term Mistreatment * proxy structural racism domain	15.667* [1.348–182.058]	0.028	10.217 [0.938–111.325]	0.056	0.916 [0.081–10.332]	0.943
Mistreatment	1.294 [0.325–5.5150]	0.714	1.972 [0.579–6.712]	0.277	6.400 [0.739–55.422]	0.092
Proxy structural racism domain	1.990* [1.027–3.855]	0.041	1.460 [0.778–2.741]	0.239	1.321 [0.677–2.580]	0.415

Included in the adj. model: community birth setting, income < $30,000, income = $30,000–$49,999, income = $50,000 = $69,999, income = $70,000–$99,999, insurance status, US born, racial concordance, doula support, and midwife provider

^*^p <.05. **p <.01. ***p <.001

## Discussion

Study findings reveal pervasive worry about pregnancy and childbirth (for themselves and members of their community) among Black women in the U.S., with 186 of 260 respondents (71.5%) responding affirmatively to the statement “Based on what I or members of my community have experienced, I am worried about our experiences of being pregnant, giving birth,” The results of our analyses demonstrate that Black women’s worry about themselves and their communities are grounded in both experiences of obstetric racism and structural racism within the perinatal health care system. Indeed, we found that experiences of obstetric racism during perinatal care, using mistreatment as a proxy in our secondary data analysis, were significantly predictive of worrying about pregnancy and birth for themselves and their community and were ultimately magnified within contexts of structural racism [50], manifested and measured as hidden resources (i.e., the unavailability of perinatal health care resources for women of color). Additionally, there is a direct effect of a context of hidden resources on worry, suggesting that manifestations of structural racism in the domain of hidden resources, as defined for, by, and with Black women, influence Black women’s worry about pregnancy and birth, independent of experiences of obstetric racism. Taken together, the structural and systemic quality and patient safety violations during perinatal care identified in this study represent reproductive injustices that specifically harm Black women [4, 16, 17, 37].

The survey item used to assess the negative societal view domain captures stereotypes that Black women depend more on welfare than other women and embody the trope of the “welfare queen,” an example of misogynoir [65]. Notably, this item also connected stereotyping specifically to structural racism within the health care system (item wording: “If a pregnant woman who is Black comes to a White doctor or midwife’s office, the office staff assumes that she is on welfare.”). In the moderator analysis shown in Table 4, there was a high magnitude adjusted odds ratio of 10.217 and borderline significance (*p* = 0.056) for the relationship between mistreatment (proxy for obstetric racism) and worry about pregnancy and birth among respondents who agreed with this statement. A statistically significant interaction between experiences of obstetric racism and exposure to structural racism in this domain may have been uncovered had the sample size been larger. A previous study examined the difference in perceptions of Black and White women who may or may not be pregnant among undergraduate students. A photo of a Black woman was more likely to be perceived as receiving public assistance compared to an image of a White woman [66]. In addition, when the participants were told that the women in the photos were pregnant, the Black woman was perceived as more likely to need public assistance to help with her child than the White pregnant woman [66]. Black women are aware of these stereotypes rooted in anti-Black gendered racism and may employ strategies such as mentioning their educational or social status when seeking perinatal care [41, 63, 67]. Davis describes these strategies as racial reconnaissance, the Herculean effort that Black women make to avoid or mitigate racist encounters [39, 41]. Davis shares an example of a Black woman seeking care for in-vitro fertilization who mentions to the medical director of the clinic that her parents were physicians, relying on her social status to avoid anticipated and actual acts of obstetric racism and racial stereotyping [41].

During the conceptualization of structural racism across the Black reproductive lifespan, Black pregnant women identified hidden resources as a domain of structural racism that adversely impacts their experiences [50]. The significant relationship between worry about pregnancy and birthing experiences and fewer options for care for pregnant women of color (i.e., hidden resources) emphasizes structural racism as a driver that amplifies worry among Black women. As shown in Table 4, there is no significant main effect of mistreatment, as a proxy for obstetric racism, and the relationship between mistreatment and worry weakens in the absence of hidden resources as a manifestation of structural racism. Chambers et al. [50] found that Black women expressed a lack of resources that prioritize Black women and their families, specifically within health education. Ultimately, our findings suggest the need to address manifestations of anti-Black racism in both clinical and societal contexts, as reported acts of obstetric racism and various forms of structural racism undoubtedly influence pregnancy-related stress, prenatal care utilization, and ultimately, birth and maternal health outcomes [63, 68].

Contrary to our expectations, when we operationalized obstetric racism as experiencing pressure during labor and delivery care, we did not find a statistically significant association with worry—neither overall nor in contexts of structural racism. The selected questions surrounding “pressure” intended to capture mistreatment, as a proxy for obstetric racism, in multiple forms during care. Pressure from healthcare staff to receive an intervention might not have been perceived as coercion if this pressure was not accompanied by Black women’s perceptions of threats, force, or verbal or physical abuse. Pressure, for example, could be interpreted as persistent communication about the risk or benefit of undertaking an intervention by a medical provider. Pressure during care may imply the need to enhance the shared decision-making process [33, 69]. However, while participants may not have experienced pressure as coercion, pressure can lead to coercive or non-consented care. A previous study using the GVtM survey dataset revealed that Black women were more likely to receive non-consented care than those identified as White, but other non-Black people of color were significantly more likely to experience pressure [36]. Thus, future research should also consider examining non-consented care as a measure of obstetric racism that might also be predictive of worry among Black women.

## Limitations

This is a secondary analysis of the GVtM survey dataset. Due to the cross-sectional nature of the one-time survey, researchers cannot draw causal conclusions about the relationships between variables. GVtM was developed by service users, clinicians, community health service leaders, providers, and researchers to capture various aspects of perinatal care in the U.S. However, the GVtM study was not designed to exclusively capture comprehensive and unique intersectional factors of Black women’s experiences using both race and gender as modes of knowledge production. The instrument was also not explicitly designed for understanding, examining, and explaining obstetric racism and structural racism along histories of anti-Black racism and eugenics across the Black reproductive lifespan, limiting our application of obstetric racism as defined by Davis [3, 4, 39–41] and structural racism across the reproductive lifespan as defined by Chambers et al. [50]. Future scholars should utilize and develop measures defined for, by, and with the Black reproducing community that include historical contexts and contemporary implications of anti-Black racism and misogynoir [65], offering a more precise and accurate interpretation and demonstration of harm enacted against Black women during perinatal care [4, 18, 37, 42] including *Black Women’s Perspectives on Structural Racism from across the Reproductive Lifespan* [50], *Obstetric Racism* [3, 39, 41] *SACKRED Birth* [19], and the PREM-OB Scale® [4, 18]. 

Another potential limitation is that the question aligned with the dependent variable of “worry” simultaneously asks about personal and community experiences. Although this question begins with the preamble: “Based on what I or members of my community have experienced,” the dataset did not include a measure of knowledge of community members’ experiences. Furthermore, the collective wording of the latter half of this item (“…I am worried about our experiences of being pregnant, giving birth”) does not allow us to distinguish respondents’ worry for themselves as an individual from their worries about the community, and whether this reported worry is related explicitly to respondents’ racial community (i.e., the Black community). However, self-interest may not be easily disentangled from community interest, particularly for Black people in the U.S., who recognize their individual “linked fate” with the well-being of the Black community as a whole [70]. Moreover, research suggests that vicarious experiences of racism can occur through observation or reports from family, close friends, and even strangers and that these can lead to emotional and psychological responses [71]. Furthermore, fear of childbirth, or tokophobia, is a clinical condition measured differently in various countries, ultimately assessing the severity of anxiety or fear surrounding pregnancy and childbirth [72]. A recent study of pregnant people in the U.S. during the COVID-19 pandemic revealed that Black mothers were more likely to report tokophobia than white women, likely connected to experiences of obstetric racism [73]. Additionally, the Generalized Unsafety Theory of Stress (GUTS) posits that an unconscious perception of unsafety can result in prolonged physiological stress responses that can be aroused in our bodies even without the presence of the threat or stressor [74]. Thus, the fear of being a Black pregnant or birthing person in the United States may also occur amid both anticipated and actual acts of obstetric racism. Future research studies should investigate how vicarious experiences with obstetric racism and other types of anti-Black racism and misogynoir—whether through family, friends, celebrities, or even fictional characters in the media—impact Black women’s fears or worries surrounding pregnancy and childbirth.

Finally, the sample of Black women included in this study was small (*n* = 260), which may have limited statistical power in the analysis, resulting in null findings for some of the hypothesized effects (e.g., note that the interaction term in the middle column of Table 4 for the relationship between experiences of obstetric racism and worry in the context of negative societal views approaches but does not reach statistical significance). The small sample of Black women also may have failed to fully represent the experiences of the overall population of Black women in the United States. For example, most of the sample gave birth in a hospital setting; however, a higher proportion of the sample gave birth in the community setting than among Black women nationwide [75]. Based on previous literature and the experiences of Black women, the research team was initially interested in analyzing mistreatment and pressure as predictors of worry conditioned on the birth setting. However, this was not feasible due to low reports of mistreatment (*n* = 3, 4.0%) and pressure (*n* = 2, 2.7%) in the community birth setting among Black respondents (Supplemental Material 3). This aligns with existing literature identifying free-standing birth centers and home births as safer pregnancy and birthing care options than hospital settings [33, 69, 75–78].

## Conclusion

In summary, this study highlights the heightened sense of worry about pregnancy and birth among Black women. Although experiencing mistreatment (i.e., a proxy of obstetric racism) during perinatal care was a significant predictor of worry, we found that the relationship between obstetric racism and worry maintained statistical significance only in contexts of structural racism manifested as “hidden resources” (contexts in which Black women agreed that “Pregnant women of color have fewer options for pregnancy and birth care.”). Based on the instrument and sample from GVtM, the findings of our secondary cross-sectional data analysis demonstrate that these worries are rationally rooted in the complex experiences of Black women navigating reproductive injustices within health structures and systems. Understanding Black women’s worries and how they are influenced by experiences of obstetric racism and structural racism is a critical first step for remedying injustices in obstetric care that uniquely and unfairly violate the right to quality care and patient safety for Black pregnant, birthing, and postpartum women. This study also highlights that white-Black comparisons (i.e., white reference groups) serve as an epistemic limitation and are not vital for researchers to measure, assess, and validate the experiences of Black women. Furthermore, scholars should continue to study the pregnancy and birth-related worries of Black women and birthing people as an outcome. Emerging research has suggested that emotional safety is synonymous with patient safety, and thus, safety from emotional harm or worry prompted by experiences of mistreatment as a proxy for obstetric racism, as illustrated in this study, is equally as important as “being safe” from physical harm [16, 21]. Additionally, freedom from obstetric racism during perinatal care is imperative to achieving reproductive justice. Only by eliminating obstetric racism and structural racism in its many forms, both in and out of the healthcare setting, will Black mothers and birthing people be free from worry about pregnancy and birthing while Black.

## Supplementary Information

Below is the link to the electronic supplementary material.

Supplementary file1 (DOCX 29 KB)

Supplementary file2 (DOCX 12 KB)

Supplementary file3 (DOCX 27 KB)

## Data Availability

Data is only available by request to The Birth Place Lab.
