# Supplementary material for: Manifestations of Anti-Black Racism and Worry About Pregnancy and Birthing While Black: A Cross-sectional Secondary Analysis of Giving Voice to Mothers
Source: J Racial Ethn Health Disparities. 2025 May 6;13(4):2856–68. doi: 10.1007/s40615-025-02461-2 (PMC13346305; doi:10.1007/s40615-025-02461-2)
Supplement: Supplementary file 1 — Supplementary file1 (DOCX 29 KB) [file 40615_2025_2461_MOESM1_ESM.docx]

Supplemental Material 1: Theoretical Framework Domains and Definitions

|  | **Dimensions of Obstetric Racism**  **(Domains & Responses)** [1–5] | **Definitions** |
| --- | --- | --- |
| 1 | Neglect, Dismissiveness, or Disrespect | When medical professionals ignore or dismiss a person's expressed need for reproductive help or care and/or treat them with disdain. |
| 2 | Medical Abuse | Can occur when medical professionals engage in experimentation and/or (repetitive) behavior that is motivated not by concern for the patient but serves to validate the clinicians' self-worth and upholds their domination over the patient. |
| 3 | Coercion | When medical professionals perform procedures without consent and/or intimidate patients to make decisions. |
| 4 | Intentionally Causing Pain | When medical professionals fail to appropriately manage pain, which  may be rooted in racialized beliefs about pain immunity and as well as the absence of empathy for Black people's physical suffering, leading to lack of internal motivation to alleviate or reduce Black suffering. |
| 5 | Ceremonies of Degradation | The ritualistic ways in which patients are humiliated or shamed and includes a sense of being sized up to determine the worthiness of the patient or their support person(s) who may be viewed as a threat. In response, medical staff may deploy security, police, social services, or psychiatry to ensure compliance or to remove the "threatening" person. |
| 6 | Diagnostic Lapse | When a clinicians' uninterrogated belief that Blackness is pathological leads them to de-emphasize or exaggerate or ignore a patient's symptoms resulting in an inappropriate or lapsed diagnosis. |
| 7 | Racial Reconnaissance | Describes the Herculean effort made by Black women to avoid or mitigate racist encounters, including being hypervigilant about procedures and finding providers. |
| 8 | Resistance | A strategy whereby a person uses the knowledge they have to push back against the mandates or requirement to intervene. It is an act of mobilization against a system. It can involve questioning the terms of intervention and negotiating various forms of involvement in decision making. It is rooted in having a vision of how one wants to be treated based on their own terms. |
| 9 | Refusal | Where one does not consent to participate in the medicalization of birthing. They deploy their own will against a system of control and the demands of consent. Refusal, in this case, is informed by a clear sense that one rejects the possibility of coercion, medical intervention, abuse, disrespect and the power that is used to dominate. It goes  beyond people understanding the implications of not doing something, but rather they understand and are concerned about agreeing to do something. |

|  | **Domains of Structural Racism across the Black Reproductive Lifespan**[6] | **Definitions** |
| --- | --- | --- |
| 1 | Negative Societal Views | Historical and contemporary negative and false representations of Black people that perpetuate damaging stereotypes |
| 2 | Housing | Systemic laws and supports used to discriminate against Black individuals and families in finding adequate housing in safe and resourceful neighborhoods |
| 3 | Medical Care | Racial inequities in access to and engagement in adequate reproductive health care |
| 4 | Law Enforcement | Racial inequities in the number and type of interactions with police officers |
| 5 | Hidden Resources | A lack of knowledge of availability of community resources and programs addressing women’s health issues |
| 6 | Employment | Unequal employment opportunities and support for Black women and families |
| 7 | Education | The educational system explicitly and implicitly designed to create and sustain racial inequities in disciplinary acts and access to resources in the school system |
| 8 | Community Infrastructure | Racial inequities in community assets and deficits |
| 9 | Policing Black Families | The direct and indirect monitoring of Black women and children in public and private spaces |

1. Davis D-A (2020) Reproducing while Black: the crisis of Black maternal health, obstetric racism and assisted reproductive technology. Reproductive Biomedicine & Society Online 11:56–64. https://doi.org/10.1016/j.rbms.2020.10.001

2. Davis D-A, Varner C, Dill LJ (2021) A Birth Story. Anthropology News

3. Davis D-A (2019) Obstetric Racism: The Racial Politics of Pregnancy, Labor, and Birthing. Medical Anthropology 38:560–573. https://doi.org/10.1080/01459740.2018.1549389

4. Davis D-A (2022) Traumatic repercussions: Black birthing people and obstetric racism. Universitat Autònoma de Barcelona (Virtual)​

5. Davis D-A (2022) A grammar of obstetric racism. University College of London (Live)

6. Chambers BD, Arega HA, Arabia SE, Taylor B, Barron RG, Gates B, Scruggs-Leach L, Scott KA, McLemore MR (2021) Black women’s perspectives on structural racism across the reproductive lifespan: A conceptual framework for measurement development. Maternal and Child Health Journal. https://doi.org/10.1007/s10995-020-03074-3
