# Supplementary material for: Manifestations of Anti-Black Racism and Worry About Pregnancy and Birthing While Black: A Cross-sectional Secondary Analysis of Giving Voice to Mothers
Source: J Racial Ethn Health Disparities. 2025 May 6;13(4):2856–68. doi: 10.1007/s40615-025-02461-2 (PMC13346305; doi:10.1007/s40615-025-02461-2)
Supplement: Supplementary file 2 — Supplementary file2 (DOCX 12 KB) [file 40615_2025_2461_MOESM2_ESM.docx]

Supplemental Material 2: Covariates

*Individual-Level Maternal and Birth Characteristics*

**Medicaid Insurance –** Respondents were asked to select their primary source of payment for all maternity care services. Responses recorded as “Medicaid or CHIP” were coded 1, zero otherwise.

**Income –** Respondents were asked to select a range that best describes their total household income before taxes last year. Responses were grouped into ranges: < $30,000/year, $30,000 - $49,999, $50,000-$69,999, $70,000-$99,999, and $100,000 or more.

**U.S. Born –** Respondents were asked if they were born in the USA. The responses were recorded as “yes” (coded 1) or “no” (coded 0).

**Doula Supported Birth –** Respondents were asked, “During labor and birth, some women get support from someone who is present to make them more comfortable and explain what is happening. Who, if anyone, gave you this type of support when you were in labor?” If the response was recorded as “a doula or trained labor assistant,” this variable is coded as 1, zero otherwise.

**Midwife for Prenatal Care -** Respondents were asked, “Which type of caregiver was most directly involved with giving you prenatal care?”. Any response indicating a midwife as the primary provider was coded as 1, zero otherwise.

**Patient-Provider Racial Concordance –** Respondents were asked if the midwife or doctor who cared for them most during their most recent pregnancy shared the same race, culture or heritage. Responses were recorded as “yes” (coded 1) or “no” (coded 0).

**Birth Setting –** Respondents were asked to select where their baby was born. The options were hospital (coded 0) or community setting (coded 1).
