# Supplementary material for: Manifestations of Anti-Black Racism and Worry About Pregnancy and Birthing While Black: A Cross-sectional Secondary Analysis of Giving Voice to Mothers
Source: J Racial Ethn Health Disparities. 2025 May 6;13(4):2856–68. doi: 10.1007/s40615-025-02461-2 (PMC13346305; doi:10.1007/s40615-025-02461-2)
Supplement: Supplementary file 3 — Supplementary file3 (DOCX 27 KB) [file 40615_2025_2461_MOESM3_ESM.docx]

Supplemental Material 3: Descriptive Characteristics by Birth Setting

*Worry about pregnancy and birthing experiences for community births by variables of interest and key covariates*

| **Worry About Pregnancy and Birthing Experiences** | | | | |
| --- | --- | --- | --- | --- |
|  | Overall | Not Concerned  n = 25  (33.3%) | Concerned  n= 50  (66.7%) |  |
|  | N= 75 |  |  | *P* Value |
| **Independent Variables** |  | n (%) | n (%) |  |
| Mistreatment during care |  |  |  | 0.211 |
| Experienced mistreatment | 3 (4.0%) | 0 (0%) | 3 (6.0%) |  |
| Did not experience mistreatment | 72 (96.0%) | 25 (100%) | 47 (94.0%) |  |
| Pressure during care |  |  |  | 0.612 |
| Experienced pressure during care | 2 (2.7%) | 1 (4.0%) | 1 (2.0%) |  |
| Did not experience pressure | 73 (97.3%) | 24 (96.0%) | 49 (98.0%) |  |
| **Structural Racism – Modifying Variables** | |  |  |  |
| Negative Societal Views/Misogynoir | |  |  | 0.252 |
| Any level of agreement | 40 (53.3%) | 11 (44.0%) | 29 (58.0%) |  |
| Any level of disagreement | 35 (46.7%) | 14 (56.0%) | 21 (42.0%) |  |
| Hidden Resources |  |  |  | 0.151 |
| Any level of agreement | 53 (70.7%) | 15 (60.0%) | 38 (76.0%) |  |
| Any level of disagreement | 22 (29.3%) | 10 (40.0%) | 12 (24.0%) |  |
| Educational Opportunity |  |  |  | 0.133 |
| Any level of agreement | 56 (74.7%) | 16 (64.0%) | 40 (80.0%) |  |
| Any level of disagreement | 19 (25.3%) | 9 (36.0%) | 10 (20.0%) |  |
| **Covariates** |  |  |  |  |
| Income |  |  |  | 0.919 |
| < $30,000  $30,000 - $49,999  $50,000 - $69,999  $70,000 – $99,999  $100,000 or more | 17 (22.7%)  11 (14.7%)  15 (20.0%)  14 (18.7%)  18 (24.0%) | 7 (28.0%)  3 (12.0%)  5 (20.0%)  5 (20.0%)  5 (20.0%) | 10 (20.0%)  8 (16.0%)  10 (20.0%)  9 (18.0%)  13 (26.0%) |  |
| Insurance |  |  |  | 0.845 |
| Medicaid | 17 (22.7%) | 6 (24.0%) | 11 (22.0%) |  |
| Private or Other | 58 (77.3%) | 19 (76.0%) | 39 (78.0%) |  |
| Born in the U.S. |  |  |  | 0.146 |
| Yes | 71 (94.7%) | 25 (100%) | 46 (92.0%) |  |
| No | 4 (5.3%) | 0 (0.0%) | 4 (8.0%) |  |
| Patient-Provider Race |  |  |  | 0.251 |
| Racial Concordance | 18 (24.0%) | 4 (16.0%) | 14 (28.0%) |  |
| Racial Discordance | 57 (76.0%) | 21 (84.0%) | 36 (72.0%) |  |
| Doula Support |  |  |  | 1.000 |
| Yes | 36 (48.0%) | 12 (48.0%) | 24 (48.0%) |  |
| No | 39 (52.0%) | 13 (52.0%) | 26 (52.0%) |  |
| Midwife Provider |  |  |  | 0.513 |
| Yes | 70 (93.3%) | 24 (96.0%) | 46 (92.0%) |  |
| No | 5 (6.7%) | 1 (4.0%) | 4 (8.0%) |  |
|  |  |  |  |  |

*Worry about pregnancy and birthing experiences for hospital births by variables of interest and key covariates*

| **Worry About Pregnancy and Birthing Experiences** | | | | |
| --- | --- | --- | --- | --- |
|  | Overall | Not Concerned  n = 49  (26.5%) | Concerned  n= 136  (73.5%) |  |
|  | N= 185 |  |  | *P* Value |
| **Independent Variables** |  | n (%) | n (%) |  |
| Mistreatment during care |  |  |  | <0.001 |
| Experienced mistreatment | 60 (32.4%) | 5 (10.2%) | 55 (40.4%) |  |
| Did not experience mistreatment | 125 (67.6%) | 44 (89.8%) | 81 (59.6%) |  |
| Pressure during care |  |  |  | 0.235 |
| Experienced pressure during care | 115 (62.2%) | 27 (55.1%) | 88 (64.7%) |  |
| Did not experience pressure | 70 (37.8%) | 22 (44.9%) | 48 (35.3%) |  |
| **Structural Racism – Modifying Variables** | |  |  |  |
| Negative Societal Views |  |  |  | 0.001 |
| Any level of agreement | 93 (50.3%) | 15 (30.6%) | 78 (57.4%) |  |
| Any level of disagreement | 92 (49.7%) | 34 (69.4%) | 58 (42.6%) |  |
| Hidden Resources |  |  |  | <0.001 |
| Any level of agreement | 128 (69.2%) | 24 (49.0%) | 104 (76.5%) |  |
| Any level of disagreement | 57 (30.8%) | 25 (51.0%) | 32 (23.5%) |  |
| Educational Opportunity |  |  |  | 0.323 |
| Any level of agreement | 131 (70.8%) | 32 (65.3%) | 99 (72.8%) |  |
| Any level of disagreement | 54 (29.2%) | 17 (34.7%) | 37 (27.2%) |  |
| **Covariates** |  |  |  |  |
| Income |  |  |  | 0.218 |
| < $30,000  $30,000 - $49,999  $50,000 - $69,999  $70,000 – $99,999  $100,000 or more | 36 (19.5%)  45 (24.3%)  25 (13.5%)  21 (11.4%)  58 (31.4%) | 6 (12.2%)  9 (18.4%)  10 (20.4%)  6 (12.2%)  18 (36.7%) | 30 (22.1%)  36 (26.5%)  15 (11.0%)  15 (11.0%)  40 (29.4%) |  |
| Insurance |  |  |  | 0.303 |
| Medicaid | 60 (32.4%) | 13 (26.5%) | 47 (34.6%) |  |
| Private or Other | 125 (67.6%) | 36 (73.5%) | 89 (65.4%) |  |
| Born in the U.S. |  |  |  | 0.282 |
| Yes | 171 (92.4%) | 47 (95.9%) | 124 (91.2%) |  |
| No | 14 (7.6%) | 2 (4.1%) | 12 (8.8%) |  |
| Patient-Provider Race |  |  |  | 0.833 |
| Racial Concordance | 47 (25.4%) | 13 (26.5%) | 34 (25.0%) |  |
| Racial Discordance | 138 (74.6%) | 36 (73.5%) | 102 (75.0%) |  |
| Doula Support |  |  |  | 0.263 |
| Yes | 61 (33.0%) | 13 (26.5%) | 48 (35.3%) |  |
| No | 124 (67.0%) | 36 (73.5%) | 88 (64.7%) |  |
| Midwife Provider |  |  |  | 0.997 |
| Yes | 68 (36.8%) | 18 (36.7%) | 50 (36.8%) |  |
| No | 117 (63.2%) | 31 (63.3%) | 86 (63.2%) |  |
